# Characterizing Immersion Pulmonary Edema (IPE): A Comparative Study of Military and Recreational Divers

**DOI:** 10.1186/s40798-023-00659-4

**Published:** 2023-11-18

**Authors:** Dorian Wolff, Olivier Castagna, Jean Morin, Henri Lehot, Romain Roffi, Arnaud Druelle, Jean-Éric Blatteau

**Affiliations:** 1 SAMU 95, Hôpital NOVO, Pontoise, France; 2 Emergency Department Begin Military Hospital (HIA Begin), Saint-Mandé, France; 3 Underwater research team–ERRSO, Military Biomedical Research Institute-IRBA, Toulon, France; 4 LAMHESS (UPR 6312, Université de Nice, Nice, France; 5 Diving Medicine Consultation Services and Hyperbaric Chamber, Ste Anne Military Hospital (HIA Ste Anne), Toulon, France; 6 France French Navy Diving School, St Mandrier, France

**Keywords:** Immersion pulmonary edema, Age, Physical exercise, Recreational dive, Military dive

## Abstract

**Background:**

Immersion Pulmonary Edema (IPE) is a common and potentially serious diving accident that can have significant respiratory and cardiac consequences and, in some cases, be fatal. Our objective was to characterize cases of IPE among military trainees and recreational divers and to associate their occurrence with exposure and individual background factors such as age and comorbidity. We conducted a retrospective analysis on the medical records and diving parameters of all patients who were treated for IPE at the Hyperbaric Medicine Department of Sainte-Anne Military Hospital in Toulon, France, between January 2017 and August 2019. In total, 57 subjects were included in this study, with ages ranging from 20 to 62 years. These subjects were divided into two distinct groups based on exposure categories: (1) underwater/surface military training and (2) recreational scuba diving. The first group consisted of 14 individuals (25%) with a mean age of 26.5 ± 2.6 years; while, the second group comprised 43 individuals (75%) with a mean age of 51.2 ± 7.5 years. All divers under the age of 40 were military divers.

**Results:**

In 40% of cases, IPE occurred following intense physical exercise. However, this association was observed in only 26% of recreational divers, compared to 86% of military divers. Among civilian recreational divers, no cases of IPE were observed in subjects under the age of 40. The intensity of symptoms was similar between the two groups, but the duration of hospitalization was significantly longer for the recreational subjects.

**Conclusion:**

It seems that the occurrence of IPE in young and healthy individuals requires their engagement in vigorous physical activity. Additionally, exposure to significant ventilatory constraints is a contributing factor, with the intensity of these conditions seemingly exclusive to military diving environments. In contrast, among civilian recreational divers, IPE tends to occur in subjects with an average age twice that of military divers. Moreover, these individuals exhibit more prominent comorbidity factors, and the average level of environmental stressors is comparatively lower.

## Key points

- In the case of young, well-trained military divers without cardiovascular pathology, we have observed that the occurrence of IPE requires the simultaneous presence of intense finning exercise and/or a high negative Static Lung Load (SSL) during immersion.
- However, among civilian recreational divers, no instances of IPE were observed in individuals under the age of 40. In civilian divers, IPE appears to be more common among those who are older than their military counterparts (Average age/medians: 51.2 ± 7.5 years) and may possess cardiovascular risk factors. Even milder environmental constraints (associated with a low negative SLL) seem sufficient to induce IPE in this particular group of divers.

## Background

Immersion Pulmonary Edema (IPE) can occur during various aquatic activities, including swimming, scuba diving, and even free diving [1]. The initial clinical manifestations of IPE are dyspnea and cough, which can later be accompanied by hemoptysis, hypoxemia, and severe cardiac dysfunction, potentially leading to drowning and fatality [2–4]. Consequently, IPE is widely recognized as a potentially life-threatening condition [5–7]. Moreover, IPE can affect both young, physically fit divers and older divers with pre-existing cardiovascular risk factors [8–10]. Given these circumstances, it is pertinent to inquire whether IPE is an unpredictable event or if there are discernible predictors for its development among divers.

During immersion, a significant volume of blood (approximately 700 mL) is immediately redistributed from the peripheral circulation to the large thoracic vessels and the pulmonary circulation, a phenomenon known as the Blood shift [11]. This redistribution leads to an increase in right cardiac preload, resulting in an approximate 30% expansion of the right heart chambers and an elevation in pressure within the pulmonary capillaries [12]. Consequently, the heightened pressure in these small blood vessels facilitates the passage of plasma and cellular components through the capillary walls into the interstitial spaces and pulmonary alveoli [2].

During physical exercise on land, cardiac output and blood pressure increase to meet the heightened demand for oxygen and nutrients by the active muscles [13]. This rise in blood pressure also affects the pulmonary vessels, leading to an elevation of pressure within the pulmonary capillaries [14]. Intense physical exercise can result in increased systemic vascular permeability, which may impact pulmonary vascular permeability as well, causing fluid leakage from the pulmonary capillaries into the interstitial spaces and alveoli [15, 16]. Furthermore, intense physical exercise can trigger an inflammatory response in the body, causing the release of inflammatory mediators [17]. These mediators can enhance vascular permeability and contribute to the development of pulmonary edema [18]. Finally, intense land physical exercise can disrupt the alveolar-capillary barrier, making it more permeable to fluids and proteins, facilitating the movement of fluid from the pulmonary capillaries into the alveoli, and thereby contributing to the formation of pulmonary edema [19, 20].

The assessment of Static Lung Load (SLL) relies on comparing the diver's intrapulmonary pressure with the pressure of the inhaled gas [2, 21]. When intrapulmonary pressure drops below the pressure of the inhaled gas, the SLL becomes negative. This means that the diver needs to create a significant transpulmonary depression to ventilate effectively, resulting in a negative pressure ventilation (NPV) scenario. NPV generates a suction force that elevates the negative pressure within the interstitial space of the lungs. This excessive negative pressure can disrupt the hydrostatic equilibrium between the pulmonary blood vessels and the surrounding tissues, leading to an excessive filtration of fluid from the blood vessels into the interstitial spaces of the lungs. It also enhances pulmonary vascular permeability and increases the permeability of the blood vessel walls, facilitating the leakage of fluid from the pulmonary capillaries into the surrounding tissues and alveoli. In essence, NPV has the potential to induce the onset of acute pulmonary edema [22–24].During immersion, the cardiopulmonary system experiences three simultaneous stresses: immersion, physical exercise, and negative pressure ventilation (NPV) [2, 25, 26].

While the scientific literature generally depicts IPE as more commonly occurring in individuals around the age of 50, it is noteworthy that within our hyperbaric medicine department, IPE has emerged as the primary cause of hospitalization among young military divers.

In summary, in light of our experience as physicians in the hyperbaric medicine department, it is evident that the incidence of IPE is not purely random. The objective of this study was to systematically characterize instances of IPE among military trainees and recreational divers, as well as to establish associations between its occurrence and various individual background factors, such as age and comorbidity.

## Methods

We conducted a retrospective review of the medical records and diving parameters of all consecutive patients over 18 years old who were diagnosed with IPE and admitted to the Hyperbaric Medicine and Diving Expertise Department of Sainte-Anne Military Hospital in Toulon, France, between January 2017 and August 2019. Patients were diagnosed with IPE if they presented with acute dyspnea during immersion and exhibited either a cough with sputum/hemoptysis and crackles/wheezing on auscultation or typical findings of IPE on chest computed tomography scan. Informed consent from the patients was waived due to the retrospective nature of this study, which utilized limited anonymous summary data. However, this study was approved by the local ethics committee (Comité de Protection des Personnes « ile de France II» n°: 21.05.05.35821 RIPH1 HPS; N° ID RCB: 2021-A01225-36).

We excluded all patients with a history and clinical evidence of pulmonary barotrauma or decompression sickness from the study.

The following data were collected: age, sex, civilian or military status, cardiovascular history (hypertension, diabetes, smoking, history of heart disease or Acute Coronary Syndrome (ACS), history of previous IPE, diving equipment used, gas mixture used, maximum depth reached, surface water temperature (obtained from the nearest meteorological station), total dive time, intense physical exertion during diving (we inquired whether divers perceived the exercise intensity as high, offering only "YES" or "NO" as response options), time from the end of the dive to the onset of symptoms, initial clinical state before and after hospitalization, treatment received during hospitalization, evolution of symptoms after 12 h of treatment, evolution of symptoms after 24 h of treatment, results of pulmonary computed tomography (CT) scan, results of cardiac ultrasound (cUS), elevation of troponin levels, blood gas hypoxemia or desaturation, and time until discharge.

IPE was classified as severe if patients showed myocardial dysfunction or hypoxemia during their hospital stay. Myocardial dysfunction was defined as an elevation of troponin levels above the standard threshold or left ventricular contractile dysfunction observed on cardiac ultrasound (preserved LVEF, considering it preserved when values exceed 50%, abnormal when below 50%, and impaired when below 45%.). Severe hypoxemia was defined as a PaO2 below 80 mmHg on blood gas analysis or, if not available, a pulse oximetry measurement below 90%.

The water temperature was categorized into three groups: cold water (below 15 °C), temperate water (between 15 and 20 °C), and warm water (above 20 °C). The time until discharge was classified into three groups: outpatient care, hospitalization for 24 h, or hospitalization for more than 24 h.

### Statistics

The data underwent statistical analysis using Prism 6 software (GraphPad Software, La Jolla, California). Continuous variables were reported as means ± SD or medians (25th to 75th percentile), depending on the data distribution determined by the Agostino & Pearson normality test. Categorical variables were presented as absolute values and percentages. Normally distributed continuous variables between both populations were compared using Mann–Whitney's unpaired test. Differences in categorical variables between the two populations were assessed using the Chi-square test with Yates correction or Fisher's exact test for small sample sizes. Statistical significance was defined as *p* < 0.05 for all tests.

## Results

The military diver group comprised 14 individuals (25%) with an average age of 26.5 ± 2.6 years, while the recreational diver group included 43 individuals (75%) with an average age of 51.2 ± 7.5 years. The two groups were primarily differentiated by age, the proportion of women, and their cardiovascular history. Additionally, they varied in terms of the respiratory equipment used and the intensity of physical exercise conducted. Table 1 provides details on the subjects and diving conditions. The pathophysiological mechanisms triggering IPE are complex, involving several factors, but two fundamental elements are consistently present: the performance of intense physical exercise and an increase in negative pressure ventilatory work (negative pressure ventilation -NPV). The insights provided by the results presented in Table 1 are valuable, as they confirm that the required magnitude of these two constraints differs depending on the age of the subjects. In young military divers, who are free of any cardiovascular pathology, a high intensity of exercise and NPV are necessary to trigger IPE. In contrast, among older divers, 28% of whom have cardiovascular risk factors, moderate intensity constraints are sufficient to provoke IPE. The study's findings underscore the necessity of a significant cardiopulmonary strain for the occurrence of IPE in young individuals.

**Table 1:** Subject and Diving Condition Characteristics

Variables	All (*n* = 57)	Military divers	Recreational divers	*p*
		(*n* = 14, 25%)	(*n* = 43, 75%)	
*Characteristics of the patients*				
Age (25–75th percentile; or ± SD) years	45 (30–55)	26.5 (± 2.6)*	51.2 (± 7.5)	< 0.0001
Male, *n* (%)	40(70)	13(93)^a^	27(63)	0.0163
Cardiovascular history, *n* (%)	13 (23)	1 (7)^a^	12 (28)	0.0439
Immersion Pulmonary Edema (IPE) history, *n* (%)	12 (21)	3 (21)	9 (20)	0.0938
*Dive Characteristics*				
Breath-hold diving, *n* (%)	14 (25)	1 (7)^a^	13 (30)	0.0406
Open circuit diving, *n* (%)	33 (58)	3 (21)^a^	30 (70)	0.007
Rebreather diving, *n* (%)	4 (7)	4 (29)^a^	0 (0)	0.0001
Surface swimming, *n* (%)	6 (11)	6 (43)^a^	0 (0)	< 0.0001
Intense physical effort, *n* (%)	23 (40)	12 (86)^a^	11 (26)	< 0.0001

Concerning age, the Agostino & Pearson normality test indicated that the age distribution for the entire group of 47 divers is not normal (*p* = 0.029). Consequently, age values for the entire group of divers are presented as medians (25th to 75th percentile). However, for each of the two diver populations studied, the age distribution follows a normal pattern (*p* = 0.322 for military divers; *p* = 0.0989 for recreational civilian divers). In these cases, age values are displayed as means ± SD. Statistical significance was determined using the Mann Whitney test for unpaired data

^*^If difference between groups was significant (*p* < 0.05)

For all other data, statistical significance was determined using the Chi-square and Fischer’ exact test

^a^If differences between groups were significant (*p* < 0.05)

The description of initial symptoms and pre-hospital management is presented in Table 2. While the populations in both groups and the onset conditions differed, the time to the onset of symptoms, the initial symptoms, and pre-hospital treatment were similar in both populations. The findings from Table 2 reveal that the timing of IPE onset and the initial symptoms are comparable in both population groups. This suggests that once the body's adaptive capacities are surpassed, the disease progression follows an age-independent kinetics. In other words, once IPE is triggered, the clinical manifestations and the course of the condition appear to be similar, irrespective of the diver's age.

**Table 2:** Description of Initial Symptoms and Pre-hospital Management

Variables	All (*n* = 57)	Military divers (*n* = 14, 25%)	Recreational divers (*n* = 43, 75%)	*p*
*Time to onset of symptoms*				
Onset during dive, *n* (%)	45 (79)	12(86)	33(77)	0.2373
Onset after the dive, *n* (%)	12 (21)	2(14)	10(23)	0.2373
Onset immediately at emersion, *n* (%)	7 (58)	0(0)	7(16)	0.0535
Onset < 1h of emersion, *n* (%)	3 (25)	0(0)	3(7)	0.155
Onset > 1h of emersion, *n* (%)	2 (17)	2(14)	0(0)	0.058
*Initial symptoms*				
Dyspnea, *n* (%)	53 (93)	14 (100)	39 (91)	0.1183
Cough, *n* (%)	52 (91)	14 (100)	38 (88)	0.0908
Sputum, *n* (%)	44 (77)	12 (86)	32 (74)	0.1918
Respiratory distress, *n* (%)	11 (19)	4 (29)	7 (16)	0.1557
Initial loss of consciousness, *n* (%)	2 (4)	0 (0)	2 (5)	0.2057
Cardiac arrest, *n* (%)	1 (2)	0 (0)	1 (2)	0.2824
*Prehospital treatment*				
Normobaric oxygen therapy, *n* (%)	41 (72)	9 (64)	32 (74)	0.2318
Non-invasive ventilation, *n* (%)	6 (11)	3 (21)	3 (7)	0.0631
Orotracheal intubation, *n* (%)	1 (2)	0 (0)	1 (2)	0.2824
Time from symptom to treatment, median (Q1–Q3), minutes	11 (0–120)	11 (0–116)	16 (0–112,5)	0.786
Time from symptom to hospital, median (Q1–Q3), minutes	132 (98–194)	103,5 (79,5–138,25)	145 (101–204)	0.875

No statistical difference between groups was observed using the Chi-square and Fisher's exact test

As anticipated, pre-hospital treatment and the promptness of medical care for the subjects are alike in both groups, which is essential for assessing the extent of cardiopulmonary consequences and the rate of remission. This enables an unbiased evaluation of the effects of IPE and a comparison of the response between young and older subjects facing this medical condition.

The results of laboratory and radiological examinations, along with the duration of hospitalization, are presented in Table 3. If all the paraclinical tests were the same in both populations, it was observed that proportionally, more military divers required outpatient care, while more civilians needed hospitalization for more than 24 h.

**Table 3:** Results of laboratory and radiological examinations and duration of hospitalization

Variables	All (*n* = 57)	Military divers (*n* = 14, 25%)	Recreational divers (*n* = 43, 75%)	*p*
*Paraclinical tests*				
Initial chest tomography showing immersion pulmonary edema lesions, *n* (%)	51 (89)	13 (93)	38 (86)	0.3174
Troponin elevation, *n* (%)	28 (49)	7 (50)	21 (48)	0.4679
Hypoxemia, *n* (%)	12 (21)	5 (36)	7 (16)	0.0607
Cardiac ultrasound showing myocardial dysfunction, *n* (%)	16 (28)	4 (29)	12 (27)	0.4808
Severe IPEs, *n* (%)	31 (54)	8 (57)	23 (52)	0.4058
*Hospitalization*				
Outpatient care, *n* (%)	14 (25)	6 (43)^a^	8 (19)	0.0335
Hospitalization 24h, *n* (%)	30 (53)	7 (50)	23 (53)	0.4102
Hospitalization > 24h, *n* (%)	13 (22)	1 (7)^a^	12 (28)	0.3272

No statistical difference between groups was observed using the Chi-square and Fisher's exact test. excepted for divers hospitalized in the outpatient care department and hospitalization durations greater than 24 h

^a^If differences between groups were significant (*p* < 0.05)

## Discussion

It is noteworthy that the two groups differ not only in their status (civilian vs. military) and, consequently, their motivation (recreational vs. professional) for engaging in scuba diving, but also in age. As a result, our study's findings suggest that the occurrence of IPE in young and healthy individuals necessitates exposure to substantial environmental constraints, primarily observed in military divers. In contrast, among civilian recreational divers, IPE manifests in individuals with an age double that of military divers. These individuals present a greater number of comorbidity factors, while the average level of environmental stress required for the onset of IPE is relatively lower.

### IPE and Female Divers

The low representation of women among military divers (7%) and higher incidence of IPE in women compared to their civilian diving population (30%) is notable. Case reports and recent studies support the higher prevalence of IPE in women [27]. This observation was substantiated by the recent study by Henckes et al. [10], which confirmed a sex-based disparity in IPE incidence among recreational divers. The study reported that 46% of those who encountered IPE were women, yielding an Odds Ratio of 2.1.

Compared to men, women have smaller lung volumes and narrower airways, leading to increased mechanical ventilatory limitations during exercise [28]. Additionally, women are more susceptible to hypoxemia, possibly due to their elevated mechanical ventilatory constraints, which may weaken the blood–gas barrier and increase susceptibility to stress-induced failures [29]. Additionally, menopausal women exhibit a significant association with stress-related cardiomyopathies, such as Takotsubo syndrome, especially in IPE cases [30–33].

### Recurrence of IPE

Remarkably, more than 20% of hospitalized IPE divers, spanning all age groups, had a prior IPE episode, affirming a recurrence rate of approximately 1 in 5, consistent with existing literature [4, 34–38]. These findings support the concept of individual susceptibility to IPE, as proposed in prior studies [39, 40].

### Type of Respiratory Apparatus Used

IPE can occur in civilians during open water swimming, referred to as swimming-induced pulmonary edema (SIPE), regardless of age, especially during events like triathlons and open water swimming competitions [41]. Nevertheless, it is crucial to emphasize that our study specifically concentrated on the occurrence of IPE among divers. The analysis of diving conditions reveals that in military divers, IPE occurs equally during open-circuit or closed-circuit dives, as well as during surface swimming. This can be explained by the fact that military divers use all types of diving breathing apparatus without distinction, and surface swimming is an integral part of their activities [21]. On the other hand, in older divers, IPE does not occur during surface swimming, which is a very rare practice in recreational diving, nor during the use of closed-circuit rebreathers, which are exceptionally used in recreational diving.

### Specific Situation of Apnea

When examining the role of breath-holding (apnea) in the occurrence of IPE, it is observed that it accounts for one-third of the cases in older civilian subjects, while only one case is observed in military subjects. Importantly, the apneas leading to IPE were not simple exercises performed by divers during their dives. Instead, these cases involved exclusively apneic individuals, most of whom were not scuba divers. This activity represents a very specific form of immersion and is recognized as a triggering factor for IPE [4]. It is a practice that is not common among military divers, which likely explains why only one subject suffered from IPE during breath-holding. Military divers are typically trained to use diving apparatus and do not regularly engage in prolonged breath-holding immersions. Therefore, the circumstances of IPE occurrence differ between military divers and those who exclusively engage in apnea as a specific activity.

### IPE and Underwater Exercise

The military personnel, regularly participate in high-intensity surface swimming sessions, particularly during their training. These exercises aim to assess individuals based on their physical abilities and maintain their fitness levels. Conversely, civilian recreational divers do not engage in such high-intensity surface swimming practices.

High-intensity surface swimming is recognized as an immersion situation that promotes the occurrence of IPE [1–3, 6]. Swimming-induced pulmonary edema (SIPE) presents with the sudden appearance of cough and shortness of breath during open water swimming, occasionally accompanied by fatigue, increased sputum production, and in some cases, coughing up blood or a combination of these symptoms [42]. SIPE predominantly affects individuals who are otherwise in good health [43].

IPE during surface swimming is primarily attributed to increased cardiac workload and pulmonary capillary pressure due to sustained physical effort.

One of the most important findings of this retrospective analysis relates to the intensity of exercise performed by divers who experienced IPE. Indeed, we observed that in 40% of cases, IPE occurred following intense physical exercise. This observation is consistent with what is described in the scientific literature [4, 9, 47]. However, our study reveals a notable difference: the concept of intense physical exercise was present in only 26% of recreational civilian divers; while, it was present in 86% of younger military divers.

### Static Lung Load and IPE

Several previous studies have thoroughly described the phenomenon of transrespiratory pressure or static lung load (SLL) [21, 44, 45] and its role in the occurrence of IPE [21]. In an immersed diver, a static negative pressure can be exerted on the respiratory system (negative SLL) due to the difference between the gas pressure delivered to the mouth and the external hydrostatic pressure at the centroid of the lung. SLL is a major determinant of physical performance in divers. When SLL is negative, the inspiratory effort increases, resulting in lower pleural and airway pressures and causing greater variations in thoracic pressure while preserving the airflow in the airways. In previous studies [21, 39, 46] we have observed that exercise with negative pressure breathing further increases inspiratory work, right ventricle loading, the imbalance between the right and left sides of the heart, as well as the rate of interstitial lung water accumulation. An altered right-to-left heart imbalance leads to the development of immersion pulmonary edema when inspiratory work is high, for example, during high-intensity swimming or SCUBA diving with negative pressure breathing settings.

**Fig. 1:**
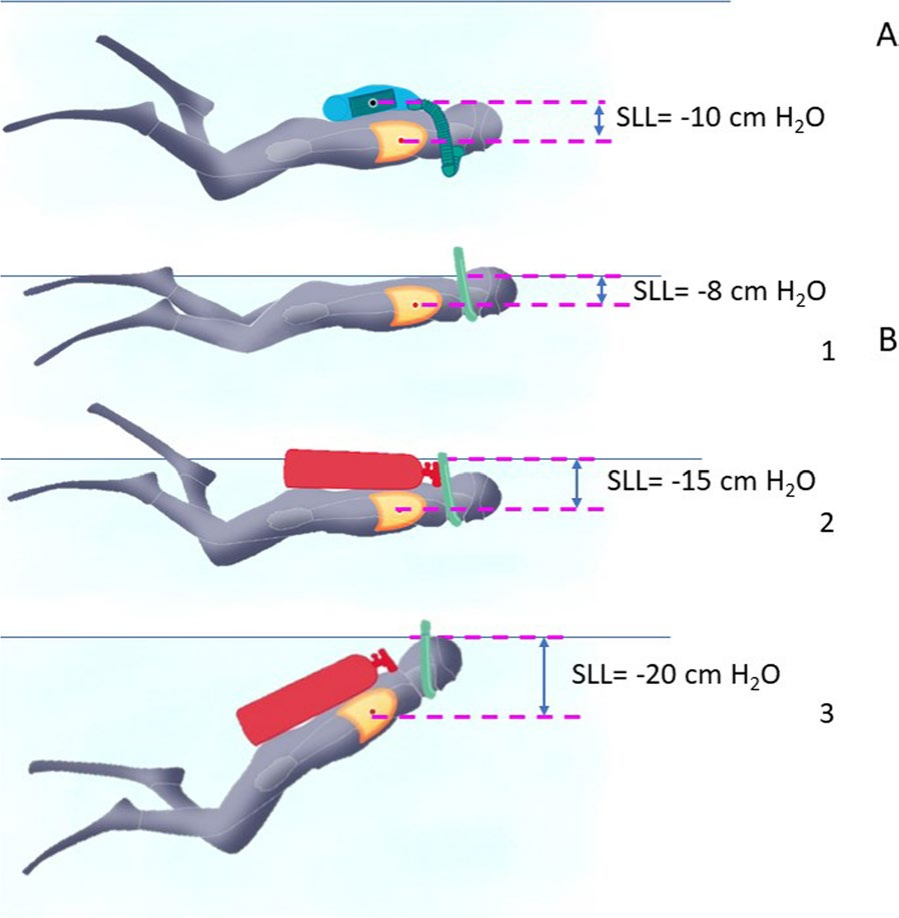
Transrespiratory pressure (Ptr) [static lung load (SLL)].** A**: a diver using a closed-circuit rebreather worn posteriorly in the prone position, the transpulmonary pressure gradient is negative (negative static lung load, SLL-).** B**(1-2-3): divers during water surface during surface fin swimming exercise, utilizing a snorkel for breathing. B-1: a diver without wearing scuba tanks. The SLL is slightly negative. B-2: a diver carrying scuba tanks. Right from the beginning of the surface exercise, the diver's torso is submerged deeper in the water, leading to a deeper negative Static Lung Load (SLL) from the onset of immersion. B-3: same diver as presented in Figure B-2 after several minutes of fin swimming. Due to fatigue, the diver gradually loses the horizontal position on the water surface and becomes less buoyant. As a result, their lower limbs start to sink, causing a tilting of the torso. This further accentuates the negative SLL value. After an hour of surface swimming, the negative SLL becomes significantly deeper compared to the beginning of the exercise

Specifically, when a diver is using a rebreather worn posteriorly in the prone position (Fig. 1A), the transpulmonary pressure gradient becomes negative (negative static lung load, SLL-). The magnitude of the SLL value varies depending on the specific rebreather model used, with values around − 10 cm H_2_O.

During surface fin swimming with the use of a snorkel, the pulmonary centroid is typically located 8–10 cm below the water level, resulting in a pressure imbalance of 8–10 cm H_2_O (Fig. 1 B1). Consequently, the diver must generate a depression of 8–10 cm H_2_O with each breath to counter this negative SLL.

In the case of surface fin swimming exercises performed by military divers, they must also carry their scuba tank on their back while breathing through a snorkel (depicted in green in the figure). In this situation, right from the start of the surface swimming exercise, the diver's torso is submerged deeper in the water. The SLL then reaches approximately 12–15 cm H_2_O (Fig. 1B2). This increases the inspiratory ventilatory effort and, consequently, the risk of IPE. During these military surface fin swimming exercises, military divers have to cover distances of several kilometers. With fatigue, gradually, the diver is no longer perfectly horizontal on the water surface and tends to stay less buoyant. Their lower limbs tend to sink, resulting in a tilting of the torso. The negative SLL value is then further accentuated. At the end of an hour of surface swimming, the negative SLL typically reaches around 18–20 cm H_2_O (Fig. 1B3). We better understand why surface swimming with a snorkel, which seems to be a low-risk exercise for divers' health, sometimes leads to the occurrence of IPE. This happens, particularly when three factors accumulate: wearing scuba tanks on the back, the intensity of the surface swimming exercise, and fatigue resulting from the duration of the activity.

It is worth noting that among military divers, nearly half of the cases of IPE occur during surface swimming. Surface swimming, performed by young military divers, is considered intense physical exercise as it is systematically timed and may lead to consequences for inadequate performance. During this exercise, divers breathe through a snorkel, which positions the centroid of their lungs between 8 and 10 cm below the water surface [21, 39]. This compels divers to generate a considerable transpulmonary pressure gradient with each breath, further intensified by the intensity of physical exercise [39]. Approximately 29% of IPE cases in young divers occur while using rebreathers, primarily due to the rebreather bag positioned on the divers' back, creating a hypostatic imbalance of at least 20 cm of water and resulting in marked NPV (Fig. 1). Additionally, 21% of IPE cases in military divers were associated with the use of open-circuit apparatus. However, in a previous study, we demonstrated that a 30 min surface swimming session with open-circuit diving apparatus, at a prescribed speed set by the French Navy, led to a significant increase in inspiratory ventilatory work, which in turn was correlated with the risk of IPE occurrence [46]. The protective role of positive SLL, in this specific circumstance, is not sufficient to counterbalance the negative effect of intense physical fin swimming exercise with open-circuit diving apparatus.

### History of Cardiovascular Pathology

Among the military subjects, only one individual had a history of cardiovascular pathology, while 28% of the civilian subjects had such a history. This difference is primarily attributed to the effect of age. Similar findings have been extensively reported in the scientific literature [4, 34–38]. In this study, all military divers where young and underwent regular medical monitoring, which enabled the exclusion of individuals potentially harboring cardiovascular risk factors before participating in their military diving practice.

Our data reported that, among young divers free of cardiovascular risk factors, IPE predominantly occurred in military divers when they were subjected to intense physical exercise and/or exposed to high negative SLL. No case of IPE was observed in young recreational divers. The results of this study could underscore the importance of subjecting young divers to significant cardiopulmonary constraints to trigger IPE.

In contrast, among older divers, the occurrence of intense physical exercise and high negative SLL is not necessary to trigger an IPE. Aging leads to a weakening of the adaptive capabilities of the cardiovascular and pulmonary systems. Additionally, some older divers may have pre-existing cardiovascular risk factors. Consequently, their bodies become more vulnerable, making them more susceptible to developing IPE even during low-intensity exercises or with less significant inspiratory ventilatory efforts.

The combined effects of immersion, physical exercise, and NPV disrupt the normal functioning of the divers' cardiopulmonary system. IPE occurs when the body's adaptation mechanisms reach their limits. Young divers have a greater capacity to adapt to more significant external constraints, which are only exceeded in the case of military divers. Conversely, in older civilian divers, even relatively moderate constraints exceed the body's adaptive capacities.

### IPE and Medical Consequences

The results presented in tables highlight an important observation: Since older civilian subjects have more fragile cardiopulmonary functions, the environmental constraints required to trigger IPE are less significant. One might have expected to observe a greater severity of cardiopulmonary impairments in older civilian subjects. However, the reality is different. Indeed, the findings from Table 3 reveal that despite the lower external constraints necessary to trigger IPE in older recreational civilian divers, the severity of observed cardiopulmonary impairments is similar to that in younger military subjects. This is demonstrated by comparable levels of troponin (a marker of myocardial injury), myocardial dysfunction on echocardiography, and "severe" IPE between the two groups. Therefore, when IPE occurs, the health consequences appear to be identical in both groups, suggesting that the health risks are similar between trained young military divers and older civilian recreational divers.

The statistical analysis indicates a significantly higher rate of hospitalization in outpatient care services among military subjects. However, it is crucial to note that this does not necessarily imply a more severe health condition among military divers. The existing protocol mandates that all military divers experiencing IPE are consistently directed to outpatient care, irrespective of symptom severity. In contrast, this standardized procedure may not always be followed by emergency services attending to civilian divers.

However, it is important to note that civilian subjects, with age-related impaired cardiopulmonary functions, exhibit a slower recovery, as evidenced by the longer duration of hospitalization. This highlights that despite experiencing similar consequences, civilian subjects have a reduced ability to recover quickly from the impairments induced by IPE compared to military subjects.

### Limitations

It is crucial to take into account the limitations and potential biases when interpreting the findings of this retrospective study. The study was conducted using data from emergency-hospitalized divers treated at the hyperbaric medicine department of military hospital in Toulon (HIA Ste Anne), which attends to a substantial number of injured divers annually, making it one of the highest figures in Europe with around 150 cases per year. However, this hyperbaric medicine department is situated in Toulon, a region with a significant presence of military divers, which could introduce a recruitment bias toward younger subjects. Due to the prominent representation of military divers, particularly those in training, the observed instances of IPE in this study may be more frequent among young subjects compared to regions without a significant military diver presence. Consequently, the generalizability of the results to the entire population of young civilian recreational divers may be limited. To gain a better understanding of the incidence and risk factors of IPE among young civilian divers, our findings should be corroborated through similar studies in regions with fewer military divers. A prospective and randomized approach, involving multiple hyperbaric medicine departments across the country, would help mitigate potential biases and produce outcomes that are more broadly applicable. Thus, it is imperative to consider these limitations when interpreting the results and to continue conducting comprehensive research to obtain a thorough understanding of the risks of IPE among young civilian divers engaged in recreational diving.

Another significant limitation of this study is related to the precise measurement of physical exercise intensity and ventilatory work. Indeed, the divers themselves subjectively determined the intensity of physical exercise. For military divers, where movement is controlled and follows a prescribed speed, estimating exercise intensity is relatively straightforward. However, for civilian recreational divers, exercise intensity depends on diving conditions, such as currents, and the diver's subjective perception. This subjectivity in estimating exercise intensity could introduce potential bias in the results. To ensure a more precise evaluation of physical effort, it would be preferable to have objective and quantifiable measures, such as heart rate or oxygen consumption. Additionally, it is crucial to note that the diver's perception can vary from person to person, making the comparison of exercise intensity between different individuals more challenging.

Concerning the extent of ventilatory work, no precise values were measured in our study. Instead, we relied on a cohort of previous scientific studies that examined ventilatory work based on the type of respiratory apparatus used [21, 39, 44, 46]. While utilizing existing data on ventilatory work can provide useful indications, individual variations and uncertainties may be associated with these estimations. Therefore, direct measurements of ventilatory work in future studies could contribute to a more accurate evaluation of this variable in the context of IPE.

#### Perspectives and Recommendations

In the realm of fin swimming and scuba diving, IPE can affect any individuals. However, it is essential to dispel the notion of inevitability when faced with IPE. Instead, identifying specific situations and risk factors that elevate the likelihood of IPE before it occurs is imperative.

Through the analysis of existing data and studies, we can pinpoint environmental constraints that favor IPE occurrence. Intense physical exercise, inspiratory effort, successive breath-hold diving along an immersed cable, and the use of certain types of diving apparatus are the primary constraints.

For civilian recreational divers aged 50 and above, it is highly advisable to avoid any strenuous physical exercise and situations that trigger an increase in negative SLL. More specifically, such divers should steer clear of vigorous fin swimming, especially in areas with strong currents. Additionally, they should refrain from utilizing respiratory systems that lead to a negative SLL, where the pressure within the lungs falls below that of the surrounding water. Figures 1 studies by Moon et al. [44] and Lundgren et al. [45], provide valuable insights into diving conditions that promote a negative hydrostatic imbalance and, consequently, an increase in NPV. It is of utmost importance that older civilian divers are made aware of these conditions and take necessary precautions to evade high-risk scenarios. By embracing these preventive measures and avoiding specific environmental constraints, older divers can minimize their risk of developing IPE and safeguard their well-being during diving activities.

Regarding young divers, our results suggests that the occurrence of IPE necessitates the combination of intense surface swimming exercise and negative SLL, constraints that are more prevalent among military divers. Consequently, young civilian divers who engage in recreational diving for leisure and pleasure are less susceptible to IPE since they are generally not exposed to these extreme constraints. Nevertheless, it is crucial to acknowledge the individuality of each person, as physiological responses may differ among divers.

## Conclusion

This retrospective analysis, involving 57 hospitalized divers with IPE, provides further evidence that physical exercise and ventilatory effort are significant external factors contributing to the occurrence of IPE. Additionally, age remains a fundamental element in the risk of developing IPE. Among military divers, a high intensity of fin swimming exercise and/or ventilatory effort is necessary for IPE to manifest. Notably, this pattern seems to be specific to military activities, as no recreational divers in this age group experienced IPE. Conversely, older civilians subjects (around 50 years old) likely display age-related impairment of cardiopulmonary functions, which explains why even milder environmental constraints can trigger IPE in this group. Moreover, it is within this older population that recovery times are notably extended.

## Data Availability

The datasets used and analyzed during the current study are available from Olivier Castagna on reasonable request.

## Abbreviations

ACS: Acute coronary syndrome
CT: Pulmonary computed tomography CT
cUS: Cardiac ultrasound
CVRF: Cardiovascular risk factors
IPE: Immersion pulmonary edema
NPV: Negative pressure ventilation
SLL: Static lung load
ULC: Ultrasound lung comets
WOB: Work of breathing
